# Diet‐induced obesity enhances the glycaemic response to glucagon in male C57BL/6J mice

**DOI:** 10.1113/EP094351

**Published:** 2026-10-25

**Authors:** Thomas H. Reynolds, Smriti Saxena, Gillian Maher, Jin Zhang

**Affiliations:** ^1^ Department of Health and Human Physiological Sciences Skidmore College Saratoga Springs New York USA

**Keywords:** glucagon resistance, glucose metabolism, insulin resistance, obesity

## Abstract

Although the impact of obesity on the action of insulin has been studied extensively, little is known about how obesity affects the action of its counter‐regulatory hormone, glucagon. The purpose of this study was to determine whether diet‐induced obesity alters in vivo glucagon responsiveness, independent of insulin action. To study diet‐induced obesity, C57BL/6J male mice were fed a normal chow diet or a Western diet for 16 weeks. Glucagon responsiveness was determined by assessing the blood glucose response to glucagon (25 µg/kg) while insulin secretion was inhibited by octreotide (1 mg/kg). Body composition was assessed by nuclear magnetic resonance spectroscopy. Insulin sensitivity was assessed by an insulin‐assisted glucose tolerance test. As expected, the Western diet resulted in an increase in adiposity and a decrease in insulin sensitivity. Contrary to our initial hypothesis, diet‐induced obesity increased glucagon responsiveness, as evidenced by significantly higher blood glucose levels following glucagon administration, resulting in a greater area under the curve in mice fed a Western diet compared with mice fed a normal chow diet. In a preclinical model of obesity and insulin resistance, our study indicates that the glycaemic response to glucagon is enhanced when insulin secretion is inhibited with octreotide, arguing against the idea of impaired glucagon responsiveness. Future studies can use this approach to identify alterations in glucagon signalling that might explain the enhanced glucose response to glucagon.

## Abstract (highlights)

- **What is the central question of this study?**
  We tested the hypothesis that pharmacologically blocking insulin secretion with octreotide before administering a maximal dose of glucagon would reveal an impaired glycaemic response to glucagon in obese mice compared with lean mice.
- **What is the main finding and its importance?**
  Contrary to our hypothesis, obesity enhanced rather than impaired the glycaemic response to glucagon. This challenges the prevailing view that obesity causes glucagon resistance with respect to glucose metabolism and suggests that enhanced glucagon action might instead contribute to the hyperglycaemia associated with obesity.

## 1 INTRODUCTION

Obesity is a major public health problem in the USA (Emmerich et al., 2024; Hales et al., 2020; Ward et al., 2019), and its prevalence has increased dramatically over the last three decades, resulting in >42.2% of adults being classified as obese (Hales et al., 2020). In 2016, the medical cost of obesity in the USA was estimated at $260 billion, a total economic burden that on an individual level corresponds to a doubling of annual medical care costs (Cawley et al., 2021). Obesity is highly associated with two metabolic diseases, type 2 diabetes (Abdul‐Ghani et al., 2024) and metabolic dysfunction‐associated steatotic liver disease (MASLD) (Sheka et al., 2020).

The impact of obesity on insulin action is well established and appears to account for the insulin resistance observed in older individuals (Basu et al., 2003; Boden et al., 1993). However, little is known about glucagon (the counter‐regulatory hormone of insulin) and its role in the development of obesity and MASLD. Glucagon is a 29‐amino‐acid peptide hormone secreted from the α‐cells of the pancreas in response to hypoglycaemia that acts primarily on the liver to increase hepatic glucose production. In addition to counteracting hypoglycaemia, glucagon stimulates hepatic lipid metabolism (Galsgaard et al., 2019) and amino acid uptake and metabolism (Albrechtsen et al., 2019; Suppli et al., 2020). Recent evidence indicates that obesity results in glucagon resistance with respect to hepatic amino acid metabolism (Albrechtsen et al., 2019; Suppli et al., 2020), but the impact of obesity on the ability of glucagon to stimulate hepatic glucose production remains unclear.

It is well established that obesity leads to an increase in hepatic glucose production, a process that is driven by hepatic insulin resistance and can lead to MASLD (Samuel & Shulman, 2016). Unlike insulin, there is a dearth of information regarding the contribution of glucagon to postprandial hyperglycaemia in obesity. Whether obesity alters the ability of glucagon to increase hepatic glucose production is not established. Obesity is associated with hyperglucagonaemia, suggestive of glucagon resistance. In the Zucker diabetic fatty rat, resistance to glucagon exists along with a decrease in the ability of insulin to reduce endogenous glucose production (Estes et al., 2024). Furthermore, reductions in the glycaemic response to glucagon and glucagon receptor expression were observed in rats fed a high‐fat diet (Charbonneau, Couturier et al., 2005; Charbonneau, Melancon et al., 2005), and mice fed a Western diet exhibited reduced glucagon signalling (Wu et al., 2024). Although these findings indicate the presence of glucagon resistance, it is important to note that insulin might have played a permissive role in the lower glycaemic response to glucagon.

In the present study, we determined whether diet‐induced obesity alters glucagon responsiveness with respect to glucose metabolism when insulin secretion is blocked with octreotide. Our hypothesis was that obesity would impair the ability of glucagon to stimulate an increase in blood glucose. To test this hypothesis, mice were administered octreotide to block insulin secretion, after which the blood glucose response to a maximal dose of glucagon was assessed. Our results indicate that glucagon responsiveness is enhanced with obesity, arguing against the idea of impaired glucagon action.

## 2 MATERIALS AND METHODS

### 2.1 Ethical approval

The study followed the guidelines set forth by the National Research Council's *Guide for Care and Use of Laboratory Animals* (2011). The experimental protocol was approved by the Skidmore College Institutional Animal Care and Use Committee (protocol #2407‐209, approved on 5 February 2024).

### 2.2 Animals

All mice were purchased from The Jackson Laboratory (Bar Harbor, ME, USA) and housed in groups of four with cage enrichment nestlets in the Skidmore College animal facility. The mice were on a 12 h–12 h light–dark cycle with the lights turning on at 08:00 h and off at 20:00 h. The temperature of the animal housing room was ∼23°C, and the humidity was 40%. Male C57BL/6J mice were purchased at 8 weeks of age and placed on a normal chow diet (NCD, *n* = 12) or a Western diet (WD, *n* = 12) for 16 weeks. The NCD and WD were purchased from Research Diets (New Brunswick, NJ, USA) and were formulated so that 10% and 60% of the kilocalories were from fat, respectively (NCD, catalogue no. D12079B; WD, catalogue no. D12492). A sensitivity power analysis (Cohen, 1988) indicated that a sample size of 12 mice per group provided 80% power (α = 0.05) to detect a between‐group effect size of Cohen's *d* = 1.20. For context, the observed glucagon responsiveness area under the curve (AUC) between‐group effect size of Cohen's *d* = 3.25. At the completion of the study, mice were killed by cervical dislocation while under general anaesthesia induced by an intraperitoneal injection of a 1:1:1 mixture of acepromzine, ketamine hydrochloride and xylazine (0.015 mL/10 g body weight).

### 2.3 Body composition

An LF50‐BCA Minispec (Bruker Inc., Billerica, MA, USA) was used to assess body composition in mice. Nuclear magnetic resonance scans were completed by immobilizing fully conscious mice in a plastic tube that was placed in the sample chamber of the instrument for ∼90 s. Body composition in mice assessed by NMR is highly correlated with the chemical analysis method of body composition assessment (Halldorsdottir et al., 2009).

### 2.4 Insulin action

The in vivo action of insulin was assessed using an insulin‐assisted glucose tolerance test (IAGTT). Following a 5 h fast, mice were injected simultaneously with glucose (2 g/kg) and insulin (0.75 U/kg). Tail vein blood (3–5 µL) was sampled 10 min before and 15, 30, 45, 60 and 90 min after the injection of glucose and insulin. The IAGTT has previously been shown to detect insulin resistance in diet‐induced obese mice (Brestoff et al., 2015; Henderson et al., 2021). Glucose was measured using a glucometer in blood collected via the tail vein (Accu‐Chek Aviva Plus, Roche Diagnostics, Indianapolis, IN, USA).

### 2.5 Glucagon responsiveness test

An overview of the glucagon responsiveness test is shown in Figure 1. Mice were fasted for 5 h, then received an intraperitoneal injection of glucagon (25 µg/kg), and tail vein blood was collected 10 min before and 5, 10, 20, 30 and 60 min after the injection. Glucose was measured using a hand‐held glucometer. To prevent the glucagon‐induced rise in blood glucose from stimulating the pancreas to release insulin, all mice received an intraperitoneal injection of octreotide (1 mg/kg) 20 min before glucagon administration. Although octreotide allows for an isolated assessment of the glycaemic response to glucagon, it creates a non‐physiological endocrine state that should be considered in the interpretation of the results.

**FIGURE 1:**
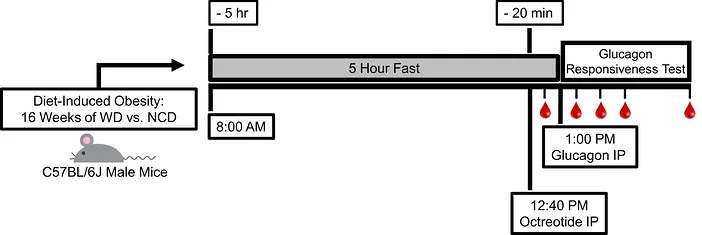
Schematic diagram of the experimental design and glucagon responsiveness test.

### 2.6 Serum insulin

Serum insulin levels were assessed by an ELISA kit from Crystal Chem (Elk Grove Village, IL, USA). To collect blood for the serum insulin assessment, NCD and WD mice from the same cohort were randomly assigned to a basal group or a glucagon group (*n* = 6 per group). The mice were subjected to the glucagon responsiveness test protocol described above, with two exceptions: mice in the basal group did not receive glucagon but instead received a vehicle injection of 0.9% saline; and 15 min after the administration of glucagon/vehicle, mice received an intraperitoneal injection of a 1:1:1 mixture of promace, ketamine hydrochloride and xylazine (0.015 mL/10 g body weight). Five minutes after injection of the anaesthetic cocktail, when the mice were completely unconscious, trunk blood was harvested and the mice were killed by cervical dislocation. The blood was allowed to coagulate at room temperature for 30 min, after which serum was isolated by centrifugation at 6000*g* for 20 min. The serum was stored at −80°C until analysis.

### 2.7 Chemicals

Glucagon was purchased from Tocris Bioscience (Bio‐Techne, Minneapolis, MN, USA), insulin from Thermo Fisher Scientific (Waltham, MA, USA), octreotide from MWI Animal Health (Boise, ID, USA) and the anaesthetic agents were purchaced from MWI Animal Health (Boise, ID, USA). All other chemicals were purchased from Sigma Chemical (St. Louis, MO, USA).

### 2.8 Statistical analysis

All statistical analyses were carried out using commercially available software (GraphPad Prism, San Diego, CA, USA). A two‐way ANOVA with repeated measures was used to compare the glucose values during the IAGTT and the glucagon responsiveness experiments between NCD and WD over time (IAGTT, 0–90 min; glucagon responsiveness test, 0–60 min). Student's unpaired *t*‐test was used to detect statistical differences in the AUC, body mass and body composition parameters between the groups. A two‐way ANOVA was used to compare basal and glucagon‐stimulated insulin levels in NCD and WD mice. Following a significant two‐way ANOVA, Fisher's LSD *post hoc* test was used to detect statistical differences in selected mean comparisons. Data are presented as the mean ± SD, and the level of statistical significance was set at *P* < 0.05.

## 3 RESULTS

### 3.1 Body composition

Body composition was assessed by nuclear magnetic resonance spectroscopy following 16 weeks of an NCD or a WD. As expected, the mice fed a WD were obese, with elevated body mass (33.58 ± 2.92 vs. 50.79 ± 2.91 g, *P* = 0.0001), total fat mass (5.82 ± 2.84 vs. 27.4 ± 1.95 g, *P* < 0.0001) and percentage body fat (18.13% ± 8.94% vs. 57.32% ± 3.16%, *P* < 0.0001) compared with mice fed an NCD. The mice fed a WD also had significantly lower lean body mass (21.7 ± 2.64 vs. 15.8 ± 1.28 g, *P* < 0.0001).

### 3.2 Insulin action

The action of insulin was assessed by an IAGTT, in which the mice received a simultaneous intraperitoneal injection of insulin and glucose, and blood glucose was measured at regular intervals for 90 min. The blood glucose values during the IAGTT were significantly different (Figure 2a; diet effect, *P* < 0.0001; time effect, *P* < 0.0001; diet × time, *P* = 0.0008). The AUCs were significantly higher in mice fed a WD compared with an NCD (Figure 2b; *P* < 0.0007).

**FIGURE 2:**
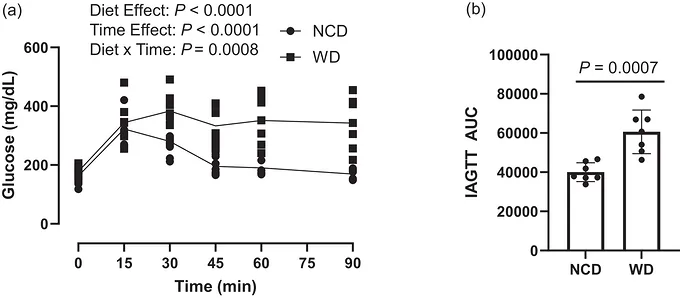
Diet‐induced obesity promotes insulin resistance. Insulin action was assessed by an insulin‐assisted glucose tolerance test (IAGTT) in mice fed a normal chow diet (NCD) or a Western diet (WD) for 16 weeks. (a) The glucose response to an intraperitoneal injection of glucose (2 g/kg) and insulin (0.75 U/kg). (b) The area under the curve (AUC) for the IAGTT. There were 12 mice per group.

### 3.3 Glucagon responsiveness

Glucagon responsiveness was assessed by administering a maximal dose of glucagon, then measuring blood glucose at regular intervals for 60 min. To prevent glucose‐stimulated insulin release, mice were pretreated with octreotide (1 mg/kg). This dose of octreotide abolishes the increase in insulin attributable to glucagon‐stimulated increases in blood glucose, allowing for the isolated assessment of the glycaemic response to glucagon (Figure 3; diet effect, *P* = 0.0087; glucagon/octreotide effect, *P* < 0.0001; diet × glucagon/octreotide, *P* = 0.0035). Furthermore, after octreotide administration the insulin levels in response to glucagon did not differ between mice fed a WD compared with an NCD (Figure 3; *post hoc* mean comparison, *P* = 0.778). In this context, glucagon responsiveness was increased significantly in mice fed a WD compared with an NCD, as evidenced by significantly higher blood glucose levels following glucagon administration (Figure 4a; diet effect, *P* < 0.0001; time effect, *P* < 0.0001; diet × time, *P* < 0.0001) and a greater AUC (Figure 4b; *P* < 0.0001). This observation demonstrates that 16 weeks of a WD increases the glycaemic response to glucagon when insulin secretion is suppressed.

**FIGURE 3:**
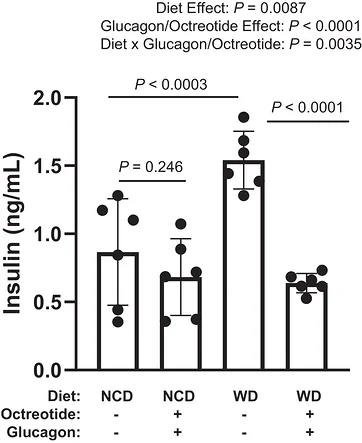
The insulin response to glucagon‐stimulated increases in glucose is blocked by octreotide. The insulin response to glucagon was assessed by administering a maximal dose of glucagon (25 µg/kg) to mice 20 min following an intraperitoneal injection of octreotide (1 mg/kg). There were six mice in each group, and blood was collected 20 min after administration of glucagon while under general anaesthesia. No statistical difference existed after glucagon administration between mice fed a normal chow diet (NCD) or a Western diet (WD), *P* = 0.778.

**FIGURE 4:**
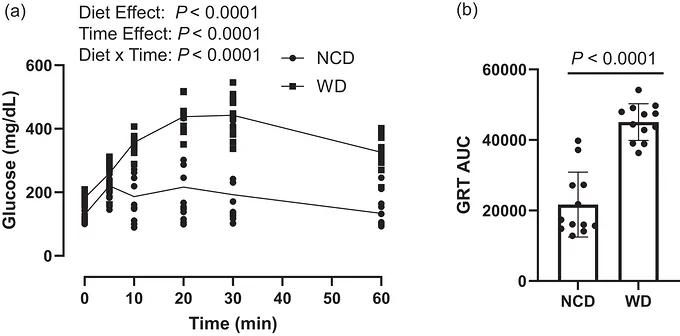
Diet‐induced obesity increases the glycaemic response to glucagon. The glucose response to glucagon was assessed by administering a maximal dose of glucagon to mice fed a normal chow diet (NCD) or a Western diet (WD) for 16 weeks. The insulin response during the glucagon responsiveness test (GRT) was blocked by an intraperitoneal injection of octreotide 20 min before glucagon administration. (a) The glucose response to an intraperitoneal injection of glucagon. (b) The area under the curve (AUC) for the GRT. There were 12 mice per group.

## 4 DISCUSSION

This study tested the hypothesis that diet‐induced obesity impairs glucagon‐stimulated increases in blood glucose. To assess directly the glycaemic response to glucagon independent of insulin, we suppressed insulin secretion pharmacologically using octreotide and measured the glycaemic response to glucagon. Using this approach, we observed that diet‐induced obesity enhances the blood glucose response to glucagon. These findings do not support the idea that glucagon responsiveness towards glucose metabolism is impaired, at least in conditions in which insulin secretion is suppressed.

It is well established that obesity leads to an increase in hepatic glucose production, a process that is driven by hepatic insulin resistance and a reduced capacity of insulin to suppress gluconeogenesis (Samuel & Shulman, 2016). Unlike insulin, a lack of information exists regarding the contribution of glucagon to postprandial hyperglycaemia in obesity. Obesity is associated with hyperglucagonaemia, suggestive of glucagon resistance, at least with respect to hepatic amino acid uptake. For example, the liver–α‐cell axis appears to explain obesity‐related hyperglucagonaemia, in which glucagon‐stimulated hepatic amino acid uptake and metabolism is impaired, raising plasma amino acid levels that stimulate the α‐cells to secrete glucagon (Albrechtsen et al., 2019; Suppli et al., 2020). Alternatively, or in concert with the liver–α‐cell axis, glucagon resistance with respect to deficits in glucagon signalling to hepatic glucose production exists in the Zucker diabetic fatty rat (Estes et al., 2024) and in rats fed a high‐fat diet (Charbonneau, Couturier et al., 2005; Charbonneau, Melancon et al., 2005; Wu et al., 2024). Our observation that the ability of glucagon to increase blood glucose is enhanced with diet‐induced obesity argues against a reduction in maximal glycaemic responsiveness to glucagon (Figure 4). In support of our findings, recent evidence suggests that diet‐induced obesity might result in overactive hepatic protein kinase A signalling following treatment with a long‐acting glucagon analogue (Long et al., 2025), and in hepatocytes from *fa*/*fa* fatty rats, glucagon‐stimulated phosphorylase activity is greater than in hepatocytes from control rats (Aiston et al., 2000). An overactive protein kinase A signal would be expected to promote greater hepatic glycogen depletion in response to glucagon. Assessing hepatic glycogen levels in conjunction with glucagon signalling might reveal a regulatory role for glycogen in the glycaemic response to glucagon. Finally, a clinical study demonstrates that the ability of glucagon to increase hepatic glucose production is not impaired in obesity, but glucagon resistance is present only for amino acid metabolism (Suppli et al., 2020), indicating a disruption in pathway‐selective glucagon signalling.

The discrepancy between our finding of enhanced glucagon responsiveness in diet‐induced obesity and findings from other preclinical studies (Charbonneau, Couturier et al., 2005; Charbonneau, Melancon et al., 2005; Estes et al., 2024; Wu et al., 2024) might reflect differences in the experimental approach. Given that the blood glucose response to glucagon stimulates insulin secretion, it is critically important to block insulin secretion to assess the action of glucagon accurately. In the present study, we suppressed the insulin response to glucagon‐induced hyperglycaemia by administering octreotide, a long‐acting somatostatin analogue that suppresses insulin secretion (Figure 3), thereby minimizing opposing effect of insulin on the glycaemic response to glucagon. Although our approach does not lead to steady‐state levels of glucagon and insulin, it is aligned with a clinical study by Suppli et al. (2020), who ‘clamped’ glucagon and insulin and reported a higher glucose response to a steady‐state elevation of glucagon.

It is important to note that our study has several limitations. One important limitation of this study is the use of octreotide to suppress insulin secretion. Although this approach allows for isolation of the glycaemic response to glucagon, it represents a non‐physiological endocrine state, in which pancreatic and pituitary gland secretions are acutely disrupted. Therefore, the enhanced glucagon responsiveness observed in the present study should be interpreted in this context and might not fully reflect the glycaemic response to glucagon in physiological conditions where insulin and growth hormone secretions are not suppressed. Nevertheless, this approach provides an important initial observation arguing against impaired glycaemic responsiveness to glucagon and might motivate future clinical studies in which steady‐state glucagon and insulin concentrations are maintained using clamp techniques. Given that it is challenging to collect liver samples in clinical studies, the of the present study can be used to study hepatic signal transduction and molecular metabolism in a preclinical obesity model. A second limitation of our study is the maximal dose of glucagon administered to the mice. Because this initial study was designed to assess maximal responsiveness, a maximal glucagon dose was used; future studies should determine whether obesity also alters glucagon sensitivity. Finally, a third limitation of the present study is the inclusion of only male mice, hence we cannot generalize our findings to females, particularly with known sex differences in the response to a high‐fat diet.

## 5 CONCLUSION

In summary, the present study provides evidence that glucagon responsiveness with respect to glucose metabolism is not impaired, but rather enhanced, in diet‐induced obesity. By suppressing insulin secretion pharmacologically, we were able to isolate the glycaemic response to glucagon and demonstrate a large difference in the blood glucose response to glucagon in obese compared with lean mice. However, it is important to note that the increase in the glycaemic response to glucagon in obesity was observed while insulin secretion was inhibited with octreotide, creating a non‐physiological endocrine state. Nonetheless, our findings challenge the idea that obesity results in glucagon resistance with respect to glucose metabolism and suggest that enhanced glucagon‐driven glucose output might contribute to the dysregulated glucose homeostasis commonly observed with obesity. Our initial findings should prompt investigations into the impact of obesity on the glucagon signalling events responsible for glycogenolysis, gluconeogenesis and lipolysis.

## AUTHOR CONTRIBUTIONS

Thomas H. Reynolds IV: conception of idea, design of study, data collection and analysis, drafting and revising manuscript. Smriti Saxena, Gillian Maher and Jin Zhang: data collection and analysis, drafting and revising manuscript. All authors approved the final version of the manuscript and agree to be accountable for all aspects of the work in ensuring that questions related to the accuracy or integrity of any part of the work are appropriately investigated and resolved. All persons designated as authors qualify for authorship, and all those who qualify for authorship are listed.

## CONFLICT OF INTEREST

None declared.

## FUNDING INFORMATION

None.

## GENERATIVE AI STATEMENT

Generative AI was used for proofreading (typographical and grammatical errors) and ensuring that the manuscript conformed to the journal's style and short communication guidelines.

## Data Availability

The data supporting the findings are available from the corresponding author upon reasonable request.
